# Automatic flow planning for fetal cardiovascular magnetic resonance imaging

**DOI:** 10.1016/j.jocmr.2025.101888

**Published:** 2025-04-01

**Authors:** Sara Neves Silva, Tomas Woodgate, Sarah McElroy, Michela Cleri, Kamilah St Clair, Jordina Aviles Verdera, Kelly Payette, Alena Uus, Lisa Story, David Lloyd, Mary A. Rutherford, Joseph V. Hajnal, Kuberan Pushparajah, Jana Hutter

**Affiliations:** a Research Department for Early Life Imaging, School of Biomedical Engineering & Imaging Sciences, King’s College London, London, UK; b Research Department for Medical Engineering, School of Biomedical Engineering & Imaging Sciences, King’s College London, London, UK; c MR Research Collaborations, Siemens Healthcare Limited, Camberley, United Kingdom; d London Collaborative Ultra high field System (LoCUS), King’s College London, London, UK; e Department of Women & Children’s Health, King’s College London, London, UK; f Smart Imaging Lab, Radiological Institute, University Hospital Erlangen, Erlangen, Germany

**Keywords:** Fetal cardiac MRI, Motion correction, Tracking, Phase-contrast

## Abstract

**Background:**

Widening access to fetal flow imaging by automating real-time planning of two-dimensional (2D) phase-contrast flow imaging (OWL).

**Methods:**

Two subsequent deep learning networks for fetal body localization and cardiac landmark detection on a coronal whole-uterus scan were trained on 167 and 71 fetal datasets, respectively, and implemented for real-time automatic planning of phase-contrast sequences. OWL was evaluated retrospectively in ten datasets and prospectively in seven fetal subjects (36+3–39+3 gestational weeks), with qualitative and quantitative comparisons to manual planning.

**Results:**

OWL was successfully implemented in 6/7 prospective cases. Fetal body localization achieved a Dice score of 0.94 ± 0.05, and cardiac landmark detection accuracies were 5.77 ± 2.91 mm (descending aorta), 4.32 ± 2.44 mm (spine), and 4.94 ± 3.82 mm (umbilical vein). Planning quality was 2.73/4 (automatic) and 3.0/4 (manual). Indexed flow measurements differed by −1.8% (range −14.2% to 14.9%) between OWL and manual planning.

**Conclusion:**

OWL achieved real-time automated planning of 2D phase-contrast cardiovascular magnetic resonance (CMR) for two major vessels, demonstrating feasibility at 0.55T with potential generalization across field strengths, extending access to this modality beyond specialized centers.

## Highlights

• Fully automatic real-time planning of 2D phase-contrast MRI scans in two of the major fetal vessels, based on a whole-uterus coronal bSSFP scan and using deep learning.
• Quantitative and qualitative evaluation of OWL: body/landmark detection, planning quality, and comparison of automated/manual flow measurements.
• Generalization to other field strengths, enabling use in diverse research and clinical settings beyond specialized centers.

## Graphical abstract

**Figure:**
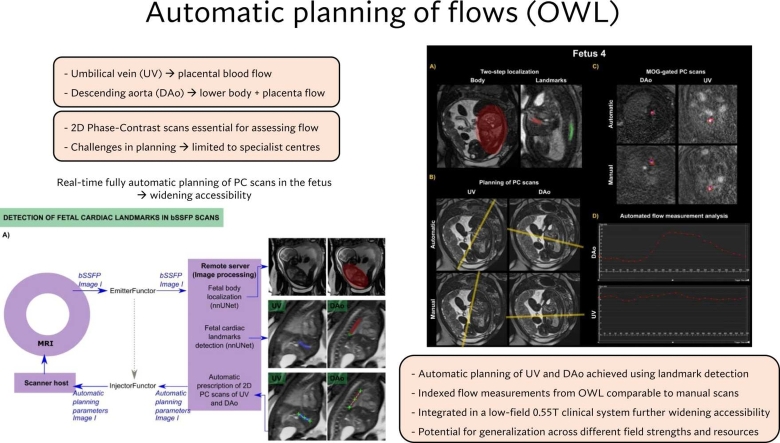

## 1 Introduction

Antenatal diagnosis complements morphological imaging by providing quantitative blood flow assessments to better evaluate fetal health. Doppler is well established for assessing major vessels such as the umbilical vein [1], uterine arteries [2] and mid-cerebral artery [3], but has limitations in flow quantification due to vessel assumptions, maternal habitus, and fetal position, particularly in late gestation. Phase-contrast cardiovascular magnetic resonance (PC-CMR) is the gold standard for postnatal hemodynamics and flow quantification [4]. Fetal two-dimensional (2D) PC flow assessment requires a cross-sectional slice to the vessel of interest, typically planned using balanced steady-state free precession (bSSFP) images of the fetal body in the axial, coronal, and sagittal orientations.

Although magnetic resonance imaging (MRI) is a standard approach for fetal brain and thoracic pathologies [5], [6], [7], it faces challenges due to the lack of conventional electrocardiogram gating and high processing demands and costs of alternative methods [8], [9]. Accurate acquisition planning remains a key challenge in fetal MRI [10], particularly for low-field MRI [11]. While low-field systems offer benefits such as improved field homogeneity, larger bore size, reduced RF heating, and lower maintenance costs [13], [12], [14], their inherently lower signal-to-noise ratio (SNR) further highlights the need for accurate planning.

Advances in AI-based real-time planning have shown promise in adult and fetal MRI, enabling automated landmark detection and acquisition plane optimization [16], [15], [17], [19], [18].

In this study, autOmatic floW planning for fetaL MRI (OWL) is proposed, combining a deep learning-based, fast cardiac landmark detection, and subsequent automated calculation of the optimal planes for the descending aorta (DAo) and umbilical vein (UV) to achieve fully automatic planning of 2D PC scans in two major vessels. Tested on ten retrospective and seven prospective fetal cardiac MR scans, OWL enables fully automated PC-MR planning, with training adaptable across field strengths to broaden accessibility beyond specialized centers.

## 2 Methods

**Fig. 1:**
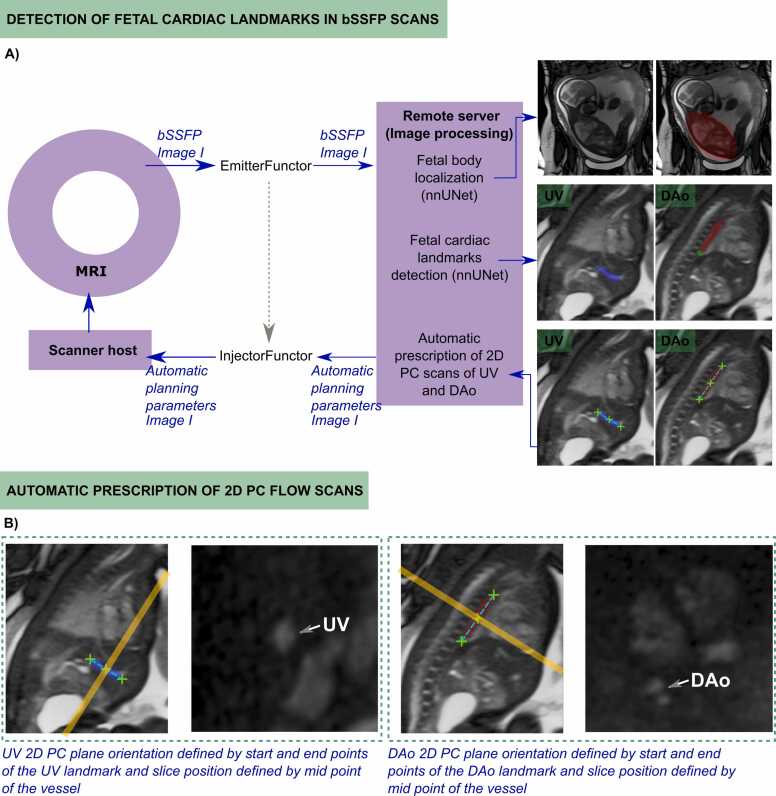
Schematic overview of the entire pipeline for automatic plane prescription. (A) Detection of the fetal cardiac landmarks in the bSSFP sequence and (B) Planning parameters applied in the 2D PC sequences. *bSSFP* balanced steady-state free precession, *2D* two-dimensional, *PC* phase-contrast

The OWL workflow (Fig. 1) involves (A) Automatic extraction of the fetal body from a whole-uterus bSSFP scan and bounding box calculation focusing on the thorax; (B) Automatic cardiac landmark detection and calculation of key points; (C) Calculation of cross-sectional plane positions and orientations for 2D PC sequences of the UV and DAo. This method was implemented on a 0.55T scanner (MAGNETOM Free.Max, Siemens Healthineers, Erlangen, Germany).

### 2.1 Fetal body localization

OWL begins with a coronal bSSFP whole-uterus scan, immediately exported to the GPU-powered remote computer (NVIDIA GEFORCE RTX 2080 Ti, NVIDIA Corporate, Santa Clara, California) for processing via the framework for image reconstruction environment [20].

A three-dimensional (3D) nnUNet [21] extracted the fetal body, and a bounding box was calculated to focus on the fetal thorax for automated cardiac landmark detection.

#### 2.1.1 Datasets, training, and testing

Fetal MRI scans were acquired on 0.55T and 1.5T clinical scanners (MAGNETOM Free.Max and Sola, Siemens Healthineers, Forchheim, Erlangen, Germany) as part of four ethically approved studies (2022–2024) at St Thomas’ Hospital (23/LO/0685, 21/LO/0742, 19LO0736, 07/H0707/105). The dataset included 167 scans (gestational ages (GA) 17–40 weeks): 114 0.55T whole-uterus Echo planar imaging (EPI) scans and 53 bSSFP scans (20 1.5T, 33 0.55T). Data augmentation with TorchIO [22] generated 1837 images, enhancing training for low SNR datasets. Testing was performed on 43 further datasets (28 0.55T EPI, 10 0.55T bSSFP, and 5 1.5T bSSFP). EPI training data was acquired at 0.55T (matrix = 128 × 128 mm, Resolution 3.1 mm^3^, TE = [46, 120, 194] ms, TR = 10.4–18.4 s, 50–55 slices, 15–30 repetitions, 4–6 min) in 114 subjects. bSSFP training data was acquired at 1.5T (Matrix = 512 × 512 mm, Resolution = 0.68 × 0.68 × 3 mm, 35 slices, TE = 2.4 ms, TR = 439.3 ms, 15 s) in 50 subjects and at 0.55T (Matrix = [288−480] × [288−576] mm, Resolution = 0.73 × 0.73 × [3–5] mm, 25–70 slices, TE = 4.0 ms, TR = 691.87 ms, 17–48 s) in 28 subjects. Prospective cases were acquired in 7 subjects at 0.55T using bSSFP (Matrix = 288 × 288, Resolution = 1.46 × 1.46 × 4 mm, 55 slices, TE = 4.22 ms, TR = 1108.58 ms, 60 s) and 2D PC (Matrix = 208 × 208, Resolution = 1.44 × 1.44 × 5 mm, TE = 5.31 ms, TR = 78.64 ms, 23 s).

### 2.2 Landmark detection

A 3D nnUNet [21] was additionally trained to extract fetal cardiac landmarks—UV, DAo, DAo endpoint at the diaphragm (DAo-D), heart-liver interface (H-L), and lung apices (AP-L). While the current pipeline focuses on UV and DAo planning, additional landmarks have been integrated to support future planning tasks (e.g., superior vena cava). Landmark coordinates were computed, transformed into the patient coordinate system, and automatically updated in the scanner host. The thoracic spine at the lung level was added to enhance DAo planning stability, aiding future pathological cases. Fig. 1B illustrates the key points extracted and used for planning the PC acquisitions.

#### 2.2.1 Datasets, training, and testing

Training included 71 (21 1.5T and 50 0.55T) cases, mainly oriented to the sagittal fetal body, with maternal coronal-oriented images added for robustness. Images were cropped to the fetal thorax and augmented [22], resulting in 1136 images. The gold standard segmentations were annotated for all cases by two fetal experts with 3–4 years of experience. The trained model was tested on 4 1.5T and 6 0.55T bSSFP datasets. Fetal body segmentation was applied first to locate the thorax for the subsequent processing steps.

### 2.3 Calculation of the 2D PC planes

#### 2.3.1 Automatic orientation and slice positioning calculation

Landmark information—start, mid, and endpoints of the UV and DAo (first two prospective cases) or UV and spine (other five cases)—was accessed by the scanner host to position and orient the 2D PC planes cross-sectional to the vessels. Orientation was based on the vector between the start and endpoints of each segmentation, and the midpoint defined the slice positioning where the indexed flow measurements would be measured (see Fig. 1B).

### 2.4 Experiments and evaluation

#### 2.4.1 Fetal body extraction and cardiac landmark detection models

Fetal body segmentation was evaluated using Dice similarity coefficient (DSC) and Intersection-over-Union (IoU) metrics against the manual annotations performed by two experts (4–6 years of experience). Cardiac landmark detection was assessed using the 3D distance between the center-of-mass (CoM) of the manual and automated segmentations.

#### 2.4.2 Real-time fetal vessel planning

Seven pregnant volunteers (GA between 36 + 3 and 39 + 6 weeks) were scanned in supine position on a clinical 0.55T MAGNETOM Free.Max scanner at St Thomas’ Hospital (June–July 2024) as part of three ethically approved studies (23/LO/0685, 21/LO/0742, 19/LO/0736). A whole-uterus coronal bSSFP scan was acquired, followed by automatically planned 2D PC scans (2D PC autoflow) of the UV and DAo using optimized parameters for 0.55T [23]. Manual planning by fetal radiographers (1–5 years of experience) was acquired for comparison. For each fetal subject, 1–3 automatic scans were acquired per vessel, and 1–2 manual acquisitions. Planning times were recorded, and quality was scored (0-poor, 1-fair, 2-optimal) for position and orientation (maximum score 4).

#### 2.4.3 Fetal blood flow measurements from automated acquisitions

From 7 subjects, 19 automated (10 UV, 9 DAo) and 17 manual (11 UV, 6 DAo) PC scans were acquired to evaluate OWL. Scans were assessed for motion artefacts and planning accuracy. Acceptable quality datasets underwent retrospective gating using metric optimized gating (MOG) [8] and flow quantification with cvi42 V5.11 (Circle Cardiovascular Imaging Inc. Calgary, Alberta, Canada). Flow measurements were indexed to estimated fetal weights, derived from automatically segmented fetal volumes [24], and compared to published reference ranges [25]. Fetal weight estimation followed the formula from [26], [25]:

(1) EFW=fetal volume (mL)*1.031+120

## Results

Retrospective and prospective results of the proposed OWL method, with performance analysis of the networks and automatic planning assessment, are presented in the following sections.

### 3.1 Extracting the fetal body

The fetal body localization model, trained on 0.55T and 1.5T datasets, achieved an overall DSC of 0.94 ± 0.05 and IoU of 0.9 ± 0.08. Segmentation took 6.68 s per volume.

### 3.2 Fetal cardiac landmark detection

The mean distance between the CoM of the landmarks in the automatic and manual segmentations was 5.77±2.91 mm, 4.32± 2.44 mm and 4.94 ± 3.82 mm for the DAo, spine, and UV, respectively. Landmark detection took 5.86 s per volume.

### 3.3 Real-time planning of PC scans of the major vessels

**Fig. 2:**
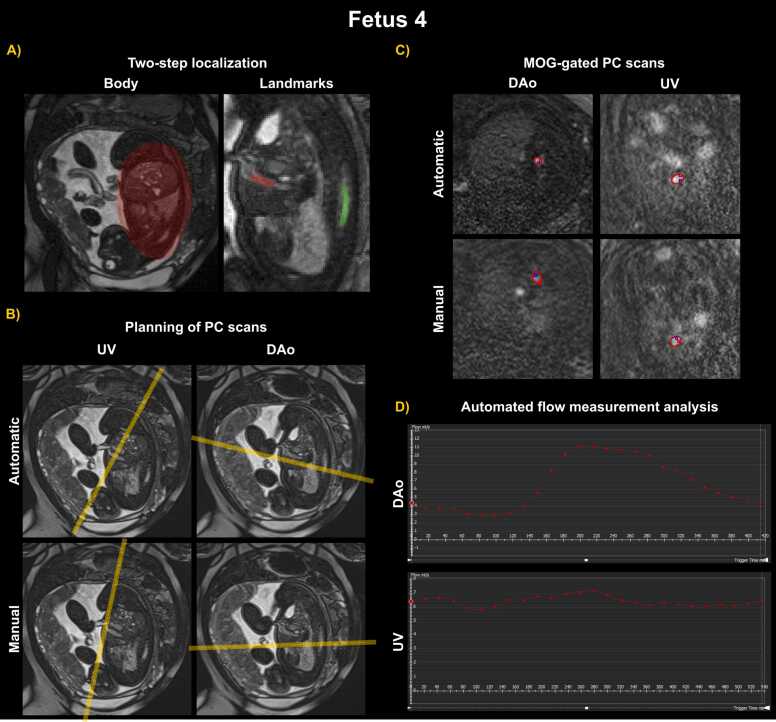
All steps of the automatic planning pipeline are shown for fetal subject 4 of the prospective dataset: (1) two-step localization of the fetal body, followed by the cardiac landmarks from which points of interest are extracted for planning (A); (2) automatic planning of the umbilical vein and descending aorta using the respective extracted landmarks (B); (3) MOG motion-corrected 2D PC scans acquired using the automatic workflow and manual planning, for comparison (C); (4) analysis of the flow measurements extracted from the automated and manual acquisitions, for validation of the developed framework (D). *MOG* metric optimized gating, *2D* two dimensional, *PC* phase-contrast

The entire pipeline was successfully run prospectively in six out of seven fetal scans. For the first two fetal subjects, the DAo and UV landmarks were used for guiding planning. In the following five subjects, the spine and UV landmarks were used. Fig. 2 depicts the entire processing pipeline for one exemplary prospective case. (A) shows the two-step localization results, (B) the automatic planning, based on the real-time detected cardiac landmarks, and matching manual planning, (C) the MOG-gated automatic and manual 2D PC acquisitions, and (D) the resulting flow measurement analysis from the gated automated scans. The estimated time between the completion of the bSSFP sequence and the start of the 2D PC sequence, ready to be run with the planning parameters correctly set for the two sequences, was 18.25 s. The average manual planning time for both sequences by a pediatric cardiologist varied between 1–2 min, and for a fetal specialist radiographer 2–3 min, although late gestation and challenging fetal positions may require an additional localization sequence that increases planning time up to 5 min.

### 3.4 Qualitative and quantitative analysis of the PC scans

The overall planning quality scores for the automatic versus manual 2D PC acquisitions, performed by a fetal specialist radiographer (automatic vs. manual), were as follows: DAo - 3.03/4 vs. 3.25/4; UV - 2.43/4 vs. 2.86/4. The overall planning quality was 2.73/4 vs. 3/4. Position and orientation were also assessed individually (automatic vs. manual), with results as follows: position - 1.36/2 vs. 1.45/2; orientation - 1.36/2 vs. 1.55/2.

From the seven prospective cases, the landmark detection task failed for subject 5 due to a challenging fetal lie unfamiliar to the network, and thus it was disregarded from any further analysis. For subjects 1 and 2, the PC sequence parameters used were not yet optimized, thus image quality was poor despite good planning (3.0/4 for each case), and MOG gating and analysis were not performed. A total of 16 PC sequences from 4 fetuses were successfully gated with MOG. Ten were acquired using automatic planning and six were manually planned. Three automatically planned sequences were not suitable for quantitative flow assessment due to the oblique intersection of the vessel of interest (two for the DAo and one for the UV). The remaining 13 PC sequences are detailed in Table 1. Planning quality assessment was additionally performed by a clinician (pediatric cardiologist with fetal experience) on the 13 MOG-gated acquisitions where flow analysis was performed (7 automatic and 6 manual) and, overall, the planning scores (automatic vs. manual) were 1.92/2 vs. 2/2.

**Table 1:** Quantitative flow data for each fetus where automated planning was applied.

Case	EFW	Vessel	Method	Flow	Indexed flow	Reference range[25]	Δ
	(kg)			(mL/min)	(mL/kg/min)	(mL/kg/min)	(%)
3	3.47	DAo	Automated	670	193	(160–344)	
			Manual	470	197		
			Automated	400	168		14.9
		DAo	Automated	457	192	(160–344)	2.8
			Manual	337	142		
4	2.38	UV	Automated	385	162	(62–206)	−14.2
		DAo	Manual	729	290	(160–344)	
			Manual	375	149		
6	2.52	UV	Automated	411	163	(62–206)	−9.6
			Manual	928	361		
		DAo	Automated	984	383	(160–344)	−6.0
			Manual	402	156		
7	2.57	UV	Automated	397	154	(62–206)	1.2

*EFW* estimated fetal weight, Δ = the percentage difference between automatically planned flow measurements and their paired manually planned measurements. Fetal subjects 3, 4, 6, and 7 of the prospective dataset were included in this analysis

*DAo* descending aorta, *UV* umbilical vein

Data are estimated fetal weight measurements (kg) for each fetal subject, calculated flow values in the respective major vessels (mL/min), flow values indexed to the estimated fetal weight measurements (mL/kg/min), reference ranges from the literature (mL/kg/min), and the percentage difference (%) between the automated and manual indexed flow mesurement for the corresponding fetus and vessel.

## 3 Discussion and Conclusion

This study presents the fully automated OWL method for planning 2D PC CMR sequences of two major fetal vessels—the umbilical vein and the descending aorta. Using a single whole-uterus coronal bSSFP scan, the method extracts cardiac landmarks for successfully planning the subsequent PC sequences orthogonal to the vessels, all in under 20 s. The method operates on the full volume and is independent of acquisition direction. The automatic scans showed comparable performance to the manual acquisitions, with no significant differences in indexed fetal flow measurements—thus reducing operator dependence and increasing time efficiency, thereby widening access to crucial information for cardiac diagnostics beyond specialist centers. Thus, while based on previously shown detection of cardiac landmarks [16], [18] and automated planning of cardiac scans [17], a novel method was successfully developed bespoke to the challenging area of fetal cardiac MRI. All processing tools were successfully implemented and prospectively tested in real-time.

The retrospective evaluation of fetal body localization and landmark extraction showed strong agreement with ground-truth segmentations performed by fetal and pediatric experts, evidenced by high DSC and IoU scores in the body extraction task, and minimal distances between the CoM of the manually segmented and automatically extracted landmarks. These results indicate sufficient robustness for the accurate calculation of the 2D PC planes. Prospective results showed notable agreement between automatic planning and manual planning in terms of planning quality and derived quantitative flow results, with significantly reduced planning time.

When scoring the adequacy of the PC sequence planning, seven automatic and six manual sequences were deemed suitable for flow analysis. The majority of indexed fetal flow measurements were within the reference range. The indexed DAo flow was slightly higher than the reference range for fetus 7, however, this finding was present in both the automated and manually planned PC sequences. When indexed fetal flow measurements were obtained from both planning methods within the same vessel, there was a mean difference in indexed flow of −1.8% (range −14.2% (162/142) to 14.9% (168/197)) with no obvious bias present in this small, initial sample. The differences observed in these cases are consistent with measurements taken within the same vessel in other published data [27], suggesting the difference in planning methodology did not lead to important differences in quantitative flow assessment.

Major strengths of the current study and the developed OWL method are the complete and robust real-time deployment, shown prospectively on seven fetuses, the speed (below 20 s), as well as the acquisition of a single bSSFP anatomical scan to visualize, extract and calculate the planes, instead of the at least three bSSFP scans in different orientations of the fetal body employed in current practice.

All steps of the OWL pipeline are available to all interested researchers (https://github.com/saranevessilva/fetal-cardiac-landmarks) and have successfully been integrated into the framework for image reconstruction environment setup [20], a key step to widen accessibility. Anonymized fetal data, both the bSSFP, the PC flow datasets, and the segmentations, are available upon request.

While the pipeline was applied here on low-field (0.55T) fetal MRI data, the OWL method is not limited to this field strength. Given the excellent results obtained even in the low-field data with low SNR, the use of 1.5T fetal MRI datasets during the training could support effective generalization to all field strengths.

There are, however, limitations. First, the OWL method was applied to a small number of fetal subjects prospectively, thus, the framework will next be applied to a larger and wider cohort and across different scanners/field strengths and pathologies. Additionally, the use of vessel-based landmarks might require significant adaptation, particularly for vessels in more challenging orientations and in congenital heart disease (CHD) pathologies where the major vessels may be of different sizes, positions, and orientations. The integration of landmarks as independent as possible from the cardiac anatomy could complement the current vessel-based landmarks as a beneficial next step. Furthermore, the study was limited to the third trimester. Motion significantly hinders the continuity between bSSFP and flow scans, especially in early GAs, a limitation further aggravated by the additional delay involved in manual planning. The OWL method, with its significantly shorter planning duration compared to the manual approach, partially mitigates the impact of motion. Optimizing the landmark detection sequence by reducing acquisition time would minimize the likelihood of fetal motion and thus improve image quality further.

This study demonstrates with OWL the feasibility of rapid, deep learning-based automatic planning for 2D PC scans of major fetal circulation vessels and introduces a framework adaptable to future extensions and improvements. This adaptability supports extending the planning to other vessels (e.g. the SVC, using the lung apices) and adapting to more complex and highly variable anatomies, such as CHD cases. Future directions involve developing a fully self-guided fetal MRI scan, where all aspects of image acquisition and analysis are automated for easy dissemination and integration of advanced and complex techniques in non-specialist centers, ultimately widening access to fetal MRI worldwide. Future work includes implementing automated quality control to evaluate acquisitions and trigger re-calculation and re-acquisition when necessary, assessing factors such as motion artefacts, anatomical coverage, and correct imaging plane orientation. Additionally, automated analysis with results available by the end of the scan will further streamline the process.

## Funding

This work was supported by the Wellcome Trust, Sir Henry Wellcome Fellowship [201374/Z/16/Z], the UKRI FLF [MR/T018119/1], DFG Heisenberg [502024488], the NIHR Advanced Fellowship [NIHR3016640], the MRC grants [MR/W019469/1] and [MR/X010007/1], and the Wellcome/EPSRC Center [WT203148/Z/16/Z]

## Author contributions

**Sara Neves Silva:** Writing – review & editing, Software, Methodology, Conceptualization. **Tomas Woodgate:** Writing – review & editing, Writing – original draft, Validation, Formal analysis, Data curation, Conceptualization. **Sarah McElroy:** Writing – review & editing, Software, Methodology, Conceptualization. **Michela Cleri:** Writing – review & editing, Validation, Formal analysis. **Kamilah St Clair:** Writing – review & editing, Validation, Formal analysis, Conceptualization. **Jordina Aviles Verdera:** Writing – review & editing, Resources, Methodology. **Kelly Payette:** Resources, Data curation. **Alena Uus:** Writing – review & editing, Software, Resources. **Lisa Story:** Writing – review & editing, Resources, Funding acquisition. **David Lloyd:** Writing – review & editing, Conceptualization. **Mary A. Rutherford:** Writing – review & editing, Resources, Funding acquisition. **Joseph V. Hajnal:** Supervision, Conceptualization. **Kuberan Pushparajah:** Writing – review & editing, Supervision, Conceptualization. **Jana Hutter:** Writing – review & editing, Writing – original draft, Supervision, Resources, Methodology, Funding acquisition, Conceptualization.

## Declaration of competing interests

The authors declare the following financial interests/personal relationships which may be considered as potential competing interests: Sarah McElroy—Siemens Healthineers.
